# Combined effect of glutamine at position 70 of HLA-DRB1 and alanine at position 57 of HLA-DQB1 in type 1 diabetes: An epitope analysis

**DOI:** 10.1371/journal.pone.0193684

**Published:** 2018-03-01

**Authors:** Petroula Gerasimou, Vicky Nicolaidou, Nicos Skordis, Michalis Picolos, Demetrios Monos, Paul A. Costeas

**Affiliations:** 1 Karaiskakio Foundation, Nicosia, Cyprus; 2 University of Cyprus, Department of Biological Sciences, Nicosia, Cyprus; 3 Department of Life and Health Sciences, School of Sciences and Engineering, University of Nicosia, Nicosia Cyprus; 4 Division of Paediatric Endocrinology, Paedi Centre for Specialized Paediatrics, Nicosia, Cyprus; 5 Alithia Endocrinology Centre, Nicosia, Cyprus; 6 Department of Pathology and Laboratory Medicine, The Children's Hospital of Philadelphia, University of Pennsylvania School of Medicine, Philadelphia, Pennsylvania, United States of America; Universita Cattolica del Sacro Cuore, ITALY

## Abstract

The contribution of specific HLA Class II alleles in type 1 diabetes is determined by polymorphic amino acid epitopes that direct antigen binding therefore, along with conventional allele frequency analysis, epitope analysis can provide important insights into disease susceptibility. We analyzed the highly heterogeneous Cypriot population for the HLA class II loci of T1DM patients and controls and we report for the first time their allele frequencies. Within our patient cohort we identified a subgroup that did not carry the DRB1*03:01-DQA1*05:01-DQB1*02:01 and DRB1*04:xx-DQA1*03:01-DQB1*03:02 risk haplotypes but a novel recombinant one, DRB1*04:XX-DQA1*03:01-DQB1*02:01 designated DR4-DQ2.3. Through epitope analysis we identified established susceptibility (DQB1 A^57^, DRB1 H^13^) and resistance (DQB1 D^57^) residues as well as other novel susceptibility residues DRB1 Q^70^, DQB1 L^26^ and resistance residues DRB1 D^70^, R^70^ and DQB1 Y^47^. Prevalence of susceptibility epitopes was higher in patients and was not exclusively a result of linkage disequilibrium. Residues DRB1 Q^70^, DQB1 L^26^ and A^57^ and a 10 amino acid epitope of DQA1 were the most significant in discriminating risk alleles. An extended haplotype containing these epitopes was carried by 92% of our patient cohort. Sharing of susceptibility epitopes could also explain the absence of risk haplotypes in patients. Finally, many significantly associated epitopes were non-pocket residues suggesting that critical immune functions may exist spanning further from the binding pockets.

## Introduction

Type 1 diabetes (T1D) is an organ-specific autoimmune disease affecting the insulin producing β cells of the pancreas, leading to absolute insulin deficiency [1]. It is one of the most common endocrine metabolic disorders in children and adolescents, however global incidence varies greatly between, and even within, countries and different ethnic populations. The lowest incidence is observed in China and the highest in Finland (0.1 v 40/ 100 000) [2] with a gradual decrease noted in countries located closer to the equator creating a north-south gradient [3]. In the Mediterranean region some areas however show notable increases such as Sardinia, which has a very high incidence similar to that of Finland, as opposed to other mainland Italian regions [4]. Great variability in T1D incidence has also been reported across different regions of Spain, where differences observed are more than threefold [5]. In the Cypriot population the incidence of T1D among children and adolescences shows an increasing trend; 10.76/100 000 between 1990–2000 [6] vs 14.9/100 000 within the subsequent five year period 2000–2004 [7]. Further studies spanning a 20-year period (1999–2009) confirm the overall increase in prevalence reporting also the largest increase in children less than five years of age [8].

Although close to 60 diabetes-associated genetic linkages have been reported to date [9], numerous studies show that the strongest association involves the inheritance of specific HLA alleles [10]. In particular, a number of HLA class II alleles namely DRB1*03:01, *04:01, *04:02, *04:05 and DQB1*02:01, *03:02 are strongly linked. A set of class II haplotypes DRB1*03:01-DQA1*05:01-DQB1*02:01 (known as DR3-DQ2.5) and DRB1*04:01/02/04/05/08-DQA1*03:01-DQB1*03:02 (known as DR4-DQ8), confer the highest risk with up to 50% of patients carrying both haplotypes, whilst haplotype DRB1*15:01-DQA1*01:02-DQB1*06:02 contributes to protection [11]. Independent associations with HLA class I alleles have also been suggested and shown to potentially affect age of onset [12–17]. More recent data have described a novel association of the less polymorphic non-classical HLA class I allele HLA-G [18–20].

HLA molecules function to present antigenic peptides to T cells and thus have a central role in immune cell activation and autoimmune disease. The peptide-binding grooves of HLA molecules are made of amino acid residues arranged in pockets; these amino acids are highly polymorphic and, either as single or groups of continuous or non-continuous residues, create millions of possible epitopes. These epitopes determine the repertoire of peptides a given HLA allele can present. Class I molecules (HLA-A, -B and -C) have binding grooves made of six pockets. Class II molecules (HLA-DR, -DQ, -DP) are heterodimers comprised of α and β chains creating binding grooves with four major pockets. In the HLA-DR heterodimer polymorphism is found only on the β chains whereas both α and β chains of the HLA-DQ and HLA-DP heterodimers are polymorphic. In addition, not all pairings between α and β chains are allowed explaining structural correlates and associations or non-associations with disease [21,22].

In addition to conventional allele frequency studies, the systematic analysis of HLA epitopes has recently been highlighted as a critical component in providing a better understanding of genetic susceptibility to T1D [23]. Indeed, even in the absence of statistically significant HLA allele association, disease susceptibility may be determined by the independent contribution of polymorphic residues participating in the formation of a functional arrangement within the binding cleft of an HLA molecule, a concept first proposed by Zerva et al. in 1996 [24]. Epitope analysis can also uncover allele associations that are missed due to their low frequency in the population or disparate alleles that share peptide-binding motifs, known as shared epitopes. In addition, single amino acid polymorphisms in the same allele have been shown to alter disease susceptibility, for example aspartic acid in position 57 of the HLA-DQB1 is protective whereas substitution with alanine is associated with susceptibility [23,25]. Amino acid differences have been shown to discriminate even closely related alleles and alter the binding avidity for insulin peptides [26]. Finally, specific epitopes may explain disease susceptibility in patients that do not carry the established risk alleles.

In the current study we investigated the HLA frequencies in a cohort of Cypriot T1D patients, a population that has not been studied to date. We propose that studying genetically heterogeneous populations such as the Cypriot population can be critically informative in allowing further validation of established risk alleles and epitopes or uncovering new ones. We report here, for the first time, the allele frequencies in Cypriot T1DM patients and a new HLA Class II risk haplotype DR4-DQ2.3. Furthermore, we identified previously reported HLA class II susceptibility residues including DQB1 L^26^ and A^57^ but also susceptibility residue DRB1 Q^70^ and alternative protective R^70^ not previously reported to be associated with T1D. Finally, we show that an extended risk haplotype of HLA class II susceptibility epitopes identified in this study DRβQ^70^-DQβL^26^A^57^DQαY^11^R^52^R^55^F^61^T^64^I^66^L^69^V/L^76^ H^129^E/K^175^ could account for 92% of our patient cohort.

## Results

### HLA class II allele frequencies in type 1 diabetes

**Fig 1:**
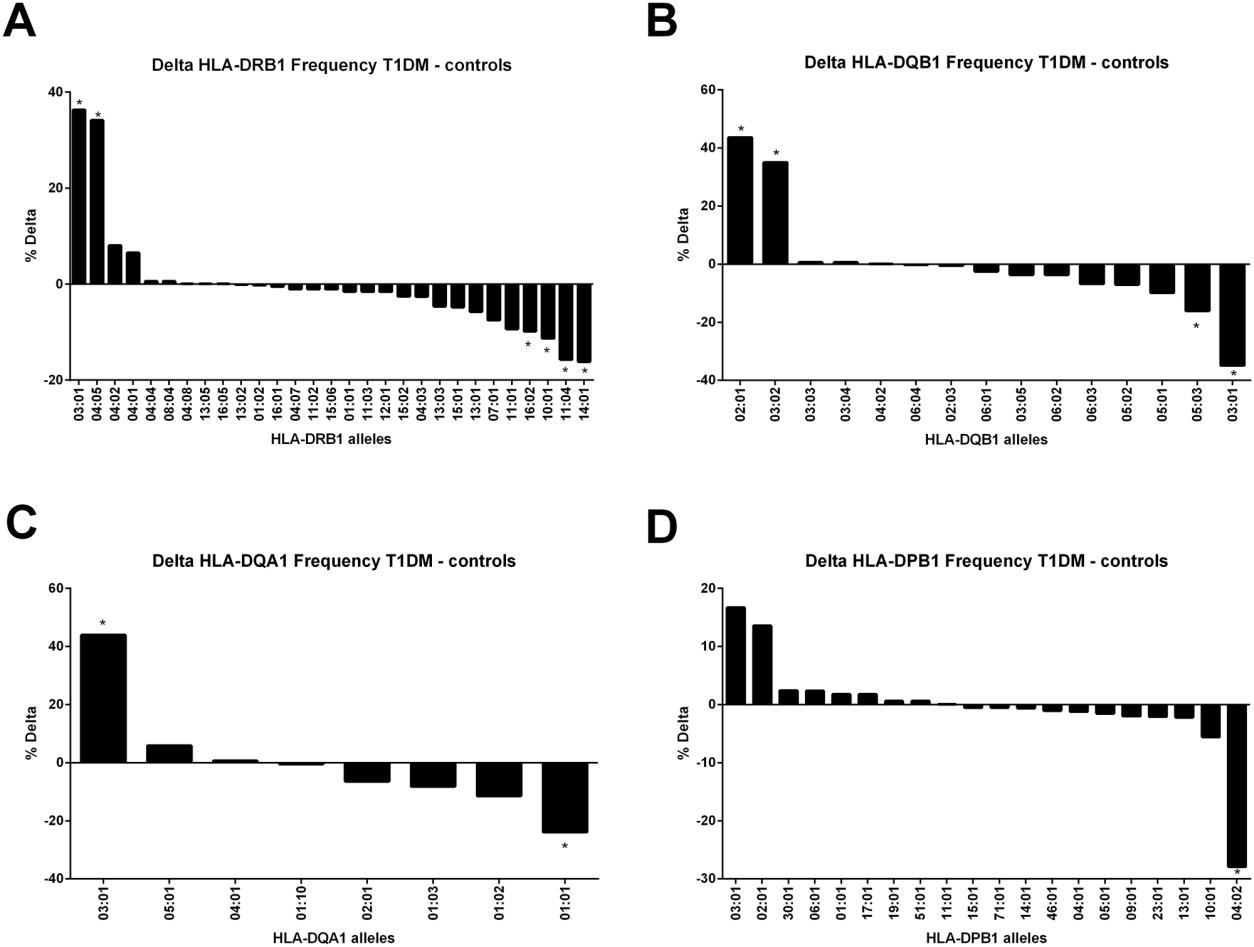
The population frequencies for HLA class II alleles. Population frequencies for HLA-DRB1 (**A**), HLA-DQB1 (**B**), HLAS-DQA1 (**C**) and HLA-DPB1 (**D**) are shown as the delta between type 1 diabetes patients and controls. The population frequency counts only how many times an allele is present in the population so in case of homozygosity it is counted as 1. * Pcorr ≤ 0.001.

We used a dataset of 170 T1D patients and 192 control subjects for whom high-resolution HLA genotyping was performed at 4 classical major histocompatibility complex class II loci DRB1, DQA1, DQB1 and DPB1. Allele frequency analysis (Fig 1 and S1–S4 Tables) showed that consistent with previous studies the alleles most significantly associated with disease were HLA-DRB1*03:01 (pcorr. = 1.06x10^-12^, OR = 6.56) and *04:05 (pcorr. = 2x10^-12^, OR = 7.28), HLA-DQB1*02:01 (pcorr. = 1.05x10^-15^, OR = 6.4) and 03:02 (pcorr. = 1.19x10^-12^ and OR = 6.58), HLA-DQA1*03:01 (pcorr = 1.76x10^-16^, OR = 6.84) and HLA-DPB1*03:01 (pcorr = 0.003, OR = 2.76). In contrast, the alleles most commonly found in the control population, and therefore deemed protective, were HLA-DRB1*14:01 (pcorr. = 4.29x10^-8^, OR = 0.02), *11:04 (pcorr. = 7.26x10^-5^, OR = 0.16), *10:01 (pcorr. = 5.06x10^-4^, OR = 0.1) and *16:02 (pcorr. = 6.77x10^-4^, OR = 0.07), HLA-DQB1*03:01 (2.85x10^-13^, OR = 0.13) and *05:03 (pcorr. = 2.14x10^-8^, OR = 0.0.2), HLA-DQA1*01:01 (pcorr. = 6.35x10^-6^, OR = 0.31) and HLA-DPB1*04:02 (pcorr. = 4.62 x10^-12^, OR = 0.07).

### HLA class II epitopes associated with susceptibility and resistance

The high-resolution HLA genotyping dataset of patients and control subjects was imported into the SKDM HLA Tool for analysis to identify epitopes associated with T1D. Both pocket and non-pocket residues were investigated for each allele and a number of them were found significantly associated. The susceptibility epitopes with the lowest p values (p ≤ 0.001) and highest OR (OR ≥ 1.5) and the resistance epitopes with the lowest p values (p ≤ 0.001) and lowest OR (OR ≤ 1.5) are summarized in Table 1. No statistically significant associations were found for HLA-DPB1 epitopes.

**Table 1:** HLA class II pocket epitopes associated with type 1 diabetes.

	Location	EPITOPE	PATIENT (N = 170)	CONTROL (N = 192)	Pcorr. Value	OR	Associated alleles
**DRB1**	13	H	110	37	8.8x10^-17^	7.57	04:05, 04:02, 04:01, 04:04, 04:08, 04:07, 04:03
	70	Q	158	97	3.6x10^-18^	12.42	03:01, 04:05, 04:01, 04:04, 04:08, 01:02, 04:07, 15:06, 01:01, 15:02, 04:03, 15:01
	70	D	84	157	7.4x10^-9^	0.22	04:02, 08:04, 13:05, 16:05, 13:02, 16:01, 11:02, 11:03, 12:01, 13:03, 13:01, 07:01, 11:01, 16:02, 11:04
	70	R	2	52	8.2x10^-12^	0.04	10:01, 14:01
	71	K	91	33	3.0x10^-11^	5.48	03:01, 04:01, 13:03
	71	R	132	185	5.12x10^-6^	0.14	04:05, 04:04, 08:04, 04:08, 13:05, 16:05, 01:02, 16:01, 04:07, 01:01, 12:01, 04:03, 07:01, 11:01, 16:02, 10:01, 11:04, 14:01
	74	R	106	24	4.2x10^-12^	6.6	03:01
	74	E	8	46	1.4x10^-5^	0.17	04:07, 04:03, 14:01
**DQA1**	11	Y	165	141	4.2x10^-9^	11	03:01, 05:01, 04:01, 02:01
	11	C	82	153	3.0x10^-8^	0.21	01:03, 01:02, 01:01
	55	R	165	141	4.2x10^-9^	11	03:01, 05:01, 04:01, 02:01
	55	G	82	153	3.0x10^-8^	0.24	01:03, 01:02, 01:01
	66	I	165	141	4.2x10^-9^	11	03:01, 05:01, 04:01, 02:01
	66	M	82	153	3.0x10^-8^	0.24	01:03, 01:02, 01:01
	69	L	165	141	4.2x10^-9^	11	03:01, 05:01, 02:01
	69	A	82	153	3.0x10^-8^	0.24	01:03, 01:02, 01:01
**DQB1**	26	L	161	92	1.4x10^-22^	18.47	02:01, 03:02, 03:03, 06:04, 02:03, 06:02, 06:03
	26	Y	17	88	1.1x10^-12^	0.14	03:04, 06:01, 03:01
	26	G	78	140	1.9x10^-5^	0.32	04:02, 03:05, 05:02, 05:01, 05:03
	47	F	121	54	1.6x10^-14^	6.2	02:01, 02:03
	47	Y	140	189	4.0x10^-6^	0.09	03:02, 03:03, 03:04, 04:02, 06:04, 06:01, 03:05, 06:02, 06:03, 05:02, 05:01, 05:03, 03:01
	57	A	160	78	1.3x10^-27^	22.3	02:01, 03:02, 03:04, 03:05
	57	D	17	121	4.5x10^-25^	0.07	03:03, 04:02, 02:03, 06:01, 06:02, 06:03, 05:03, 03:01
	70	R	166	145	1.6x10^-8^	12.1	02:01, 03:02, 03;03, 03:04, 06:04, 02:03, 06:01, 03:05, 03:01
	70	G	77	146	1.8x10^-7^	0.26	06:02, 06:03, 05:02, 05:01, 05:03

The strongest association was shown for the previously reported HLA-DQB1 residue A^57^ (p = 1.3x10^-27^, OR 22.3). This is found in HLA-DQB1*02:01 and *03:02 alleles which were significantly more common in patients according to the allele frequency analysis (S2 Table). Aspartic acid in the same position (D^57^) was identified as the residue most strongly associated with resistance (p = 4.5x10^-25^, OR 0.07). Two of the HLA-DQB1 alleles (*05:03, *03:01) sharing this residue were significantly more frequent in controls. Similarly, the presence of leucine (L) in position 26 was the second strongest residue associated with susceptibility (p = 1.4x10^-22^, OR 18.47), whilst tyrosine (Y) (p = 1.1x10^-12^, OR 0.14) or glycine (G) (p = 1.9x10^-5^, OR 0.32) at the same position confer resistance. L^26^ is found in HLA-DQB1*02:01 and *03:02 alleles which are more common in patients. HLA-DQB1 susceptibility residues F^47^ (p = 1.6x10^-14^, OR 6.2) and R^70^ (p = 1.6x10^-8^, OR 12.1) were also found in the same position as resistance residues Y^47^ (p = 4x10^-6^, OR 0.09) and G^70^ (p = 1.8x10^-7^, OR 0.26). In contrast, resistance residues A^13^ (p = 1.1x10^-12^, OR 0.14), Y^37^ (p = 1.9x10^-6^, OR 0.08), T^28^ (p = 4x10^-6^, OR 0.09) were not in the same position as susceptibility ones.

The DRB1 locus contained the next most significant HLA residue associated with susceptibility to T1D; HLA-DRB1 Q^70^ had the strongest association (p = 3.6x10^-18^, OR 12.42). This residue is shared between most high risk alleles including DRB1*03:01, *04:05 and *04:01, which were all significantly more common in patients (S1 Table). In addition, previously reported HLA-DRB1 residues H^13^ (p = 8.8x10^-17^, OR 7.57) and K^71^ (p = 3.x10^-11^, OR 5.48) were also significantly associated with susceptibility. HLA-DRB1 H^13^ is shared by a number of HLA-DRB1 *04:xx alleles that confer high risk. HLA-DRB1 *04:01 which shares all three of these epitopes had the highest OR 10.07. HLA-DRB1 resistance residues R^70^ (p = 8.2x10^-12^, OR 0.04) or D^70^ (p = 7.4x10^-9^, OR 0.22) and R^71^ (p = 5.12x10^-6^, OR 0.14) are at the same positions as susceptibility counterparts. Resistance residue R^70^ in particular had the lowest OR and is shared among two protective alleles HLA-DRB1*10:01 and *14:01 which were significantly less common in patients. The presence of arginine (R) at position 74 is associated with susceptibility (p = 4.2x10^-12^, OR 6.6), whereas glutamic acid (E) at the same position is significantly associated with resistance (p = 1.4x10^-5^, OR 0.17). Finally, residues R^30^ and A^38^ were also significantly associated with resistance and had the next lowest OR after R^70^ (p = 0.0003, OR 0.07).

A number of DQA1 residues were strongly associated with T1D; Y^11^, R^55^, I^66^ and L^69^ (p = 4.2x10^-9^, OR 11) and the previously reported R^56^ and V^76^ (p = 1.6x10^-15^, OR 6.8 (S7 Table). All are present in DQA1*03:01 (R^56^ and V^76^ are only found in this allele) most frequently observed in patients (S3 Table). Furthermore, all resistance HLA-DQA1 residues G^55^, C^11^, M^66^, A^69^, G^56^ and M^76^ (p = 3x10^-8^, OR 0.24) are at the same positions as susceptibility counterparts and are shared between HLA-DQA1* 01:03 and *01:01 alleles, which were significantly more frequent in controls.

### HLA epitopes associated with susceptibility and resistance show gene-dose effect

The effect of homozygosity or heterozygosity of class II residues identified to be associated with susceptibility or resistance was analyzed. Homozygosity of susceptibility epitope DQB1 A^57^ had the strongest association with T1D (p = 1.25x10^-24^, OR 100.6) (Table 2), whilst inheritance of two copies of the resistance residue at the same position, DQB1 D^57^, had the strongest negative association (p = 3.94x10^-9^, OR 0.03). A gene dose effect was apparent since inheritance of one copy of A^57^ decreased the probability of disease as shown by the lower OR (p = 5.61x10^-23^, OR 17.5), in the same way inheritance of one copy of the susceptibility residue D^57^ (p = 4.8x10^-22^, OR 0.08) was not as protective as inheritance of two copies but still lowered probability of disease compared to inheritance of one copy of A^57^. However, resistance appears to be dominant since inheritance of only one copy of D^57^ still conferred a lower probability of diabetes (OR 0.08). A similar effect was observed for DQB1 L^26^, inheritance of two copies is strongly associated with T1D (p = 4.3x10^-21^, OR 45.8), inheritance of one copy greatly decreased the probability of disease (p = 3.9x10^-19^, OR 14.9) and two copies of G^26^ is protective (p = 8.68x10^-6^, OR 0.11). Inheritance of two copies of the HLA-DRB1 Q^70^ susceptibility residue was also strongly associated with T1D (p = 1.4x10^-17^, OR 20.1) and probability decreased with inheritance of one copy (p = 5x10^-15^, OR 10.2). Among the two alternative resistance residues identified for this position, inheritance of R^70^ appears to be more protective than inheritance of D^70^; one copy of R^70^ conferred lower probability of disease (p = 9.44x10^-13^, OR 0.04) than one (p = 1.8x10^-7^, OR 0.27) or two copies of D^70^ (p = 6.1x10^-10^, OR 0.11). The probability of disease in the case of inheritance of two copies of R^70^ was not calculated as this genotype was not present in the population.

**Table 2:** Homozygous and heterozygous inheritance of shared HLA class II epitopes associated with type 1 diabetes.

Locus	Genotype	P corr value	OR
**HLA-DRB1**	Q^70+^/Q^70+^	1.4x10^-17^	20.1
	Q^70+^/Q^70-^	5.0x10^-15^	10.2
	D^70+^/D^70-^	1.8x10^-7^	0.27
	D^70+^/D^70+^	6.1x10^-10^	0.11
	R^70+^/R^70-^	9.44x10^-13^	0.04
	K^71+^/K^71-^	2.6x10^-12^	5.5
	R^71+^/R^71-^	4.7x10^-5^	0.2
	R^71+^/R^71+^	3.9x10^-9^	0.1
	E^74+^/E^74-^	3.4x10^-7^	0.16
**HLA-DQB1**	L^26+^/L^26+^	4.3x10^-21^	45.8
	L^26+^/L^26-^	3.9x10^-19^	14.9
	G^26+^/G^26+^	8.68x10^-6^	0.11
	A^57+^/A^57+^	1.25x10^-24^	100.6
	A^57+^/A^57-^	5.61x10^-23^	17.5
	D^57+^/D^57-^	4.8x10^-22^	0.08
	D^57+^/D^57+^	3.94x10^-9^	0.03
	R^70+^/R^70+^	1.3x10^-10^	17.6
	R^70+^/R^70-^	2.8x10^-8^	10.4
	G^70+^/G^70-^	1.3x10^-6^	0.31
	G^70+^/G^70+^	2.3x10^-8^	0.08
**HLA-DQA1**	L^69+^/L^69+^	1.07x10^-12^	22.86
	L^69+^/L^69-^	2.69x10^-8^	8.82

### Linkage disequilibrium between DQ and DR susceptibility epitopes

The prevalence of having both the highest susceptibility residues DRβ Q^70^ and the DQβ A^57^ was compared between patients and control subjects (Table 3). The majority of patients (90%) had both epitopes in contrast to 29.7% of the control subjects (p≤0.0001, OR 21.3). A very small percentage of patients had only the DRβ Q^70^ epitope (2.9%) or only the DQβ A^57^ epitope (4.1%) or none of the two epitopes (2.9%). Among control subjects the highest percentage (38.5%) had none of these susceptibility epitopes (p≤0.0001, OR 0.05). These findings suggest that our observations are not only due to linkage disequilibrium (LD) between the two loci. In addition, it was shown that these DR/DQ susceptibility epitopes were not in LD in patients (p = 0.001) but were in LD in controls (p = 1.8x10^-6^).

**Table 3:** Linkage disequilibrium of DR/DQ susceptibility epitopes in type 1 diabetes patients and control subjects.

HLA class II susceptibility epitopes	Patients (%) (N = 170)	Control subjects (%) (N = 192)	P Value	OR (95% CI)	LD in patients P value (OR)	LD in controls P value (OR)
DRβ Q^70+^/DQβ A^57+^	153 (90)	57 (29.7)	≤ 0.0001	21.3 (11.8–38.4)	0.001 (20.5)	1.80x10^-6^ (4.9)
DRβ Q^70+^/DQβ A^57-^	5 (2.9)	40 (20.8)	≤ 0.0001	0.1 (0.04–0.3)		
DRβ Q^70-^/DQβ A^57+^	7 (4.1)	21 (10.9)	0.02	0.35 (0.14–0.84)		
DRβ Q^70-^/DQβ A^57-^	5 (2.9)	74 (38.5)	≤ 0.0001	0.05 (0.02–0.12)		

n (%) p value Fisher exact test

### Shared susceptibility residues account for type 1 diabetes in patients lacking HLA class II risk haplotypes

Having identified a number of susceptibility and resistance residues associated with T1D we sought to further dissect our patient cohort with regards to their HLA genotypes aiming to explain disease susceptibility by the presence of shared susceptibility residues. More specifically, among our T1D patients the majority (135 of 170 or 79%) carried at least one or both of the susceptibility alleles DR3-DQ2.5 and DR4-DQ8 (Table 4). However, a significant number of patients (35 of 170 or 21%) did not carry any copies of either haplotype. We observed a new recombinant haplotype DRB1*04:XX-DQA1*03:01-DQB1*02:01, henceforth named DR4-DQ2.3, dominant within this subgroup; 22 (63%) of 35 patients carrying non-risk haplotypes and 13% of all patients carried one copy of the DR4-DQ2.3 haplotype in contrast to only 9 of 192 control subjects (4.7%). Interestingly, no DR4-DQ2.3 homozygous individuals were identified. The DR4-DQ2.3 haplotype contains the HLA-DQB1*02:01 allele instead of the HLA-DQB1*03:02, however both alleles share a number of DQB1 susceptibility residues such as L^26^, R^70^, L^85^, E^86^, T^89^, but more importantly the most significant susceptibility residue A^57^. Hence the presence of either HLA-DQB1*02:01 or HLA-DQB1*03:02 makes the patient homozygous for the A^57^ residue, associated with the highest OR in the zygosity analysis (p = 1.25x10^-24^ OR 100.6).

**Table 4:** HLA class II genotypes of type 1 diabetes patients and control subjects.

HLA class II Genotype	Patients (%) (N = 170)	Control subjects (%) (N = 192)	P value	OR
DR3—DQ2.5 / DRX	30 (17.65)	22 (11.46)	0.1	1.66
DR3—DQ2.5 / DR3—DQ2.5	18 (10.59)	0	<0.0001	46.7
DR3—DQ2.5 / DR4—DQ2.3	5 (2.94)	1 (0.52)	0.1	5.79
DR4—DQ8 / DRX	43 (25.29)	18 (9.38)	<0.0001	3.27
DR4—DQ8 / DR4—DQ8	5 (2.94)	1 (0.52)	0.1	5.79
DR4—DQ8 / DR4—DQ2.3	4 (2.35)	0	0.047	10.41
DR3—DQ2.5 / DR4—DQ8	30 (17.65)	1 (0.52)	<0.0001	40.93
DR4—DQ2.3 / DRX	22 (12.94)	8 (4.17)	0.004	3.42
DRX / DRX (non-risk)	13 (7.65)	141 (73.44)	<0.0001	0.03

DR3—DQ2.5 = DRB1*03:01-DQA1*05:01-DQB1*02:01, DR4—DQ8 = DRB1*04:XX-DQA1*03:01-DQB1*03:02, DR4—DQ2.3 = DRB1*04:XX-DQA1*03:01-DQB1*02:01

n (%) p value Fisher exact test

Carrying at least one copy of any of the risk haplotypes DR3-DQ2.5, DR4-DQ8 or DR4-DQ2.3 could account for 92% (157 of 170) of patients (Table 4). However, an additional 8% of our patients (13 of 170) do not carry any of the risk haplotypes. We were able to verify that all these patients carried susceptibility associated residues identified in this study in the DRB1 locus and one or more in the DQA–DQB loci.

### HLA epitopes associated with susceptibility and resistance and their potential function

To investigate whether epitopes differentiate associated alleles, allele sequences were retrieved and aligned using the IMGT/HLA database of the European Bioinformatics Institute. Within these sequences we noted all the susceptibility and resistance residues, both pocket and non-pocket, identified in this study and also found to have a proposed function according to literature [27–29]. The DQA, DQB and DRB domains show a considerable number of polymorphisms that are mainly involved in antigen binding by the anchoring pockets, the heterodimer formation by salt bridges, T-cell receptor (TCR) or CD4 co-receptor binding and in the formation of the dimer of heterodimers.

HLA DRB1 E^9^, V^11^, H^13^, Y^26^, N^37^ and R^74^ that are associated with diabetes are amino acids that are part of binding pockets (Table 5). The residue at position 57 is involved in pocket 9 but also participates in hydrogen bond formation to the peptide. A serine in that position is highly associated to T1D while an alanine is associated to susceptibility. The residues at positions 67, 70 and 71 are also part of the pocket formation but are also sites for TCR contact; alternative residues showed either susceptibility or resistance. Lastly, position 112 has a potential function in the homodimer of heterodimers and the residue at position 140 is a potential contact side for the CD4 co-receptor. HLA DQB1 residues involved in the formation of the peptide pocket include positions 13, 26, 28, 30, 37, 47, 57, 67, 70, 71, 74, 85, 86, 89 and 90 (Table 6). Amino acids at position 30 and 57 are also involved in the formation of a hydrogen bond to the peptide while residues at position 67, 70 and 71 are also a potential TCR contact site. Amino acids 52, 53 and 55 act as a homodimerization patch in the dimer formation. Pocket residues of the HLA DQA1 molecule include positions 11, 52, 66, 69 and 76 (Table 7). Amino acids at positions 69 and 76 also form a hydrogen bond to the peptide. Important residues at positions 55–64 are potential TCR contact sites while position 129 is a potential CD4 contact site. The residue at position 175 upholds a function in the formation of the homodimer of heterodimers.

**Table 5:** Type 1 diabetes associated polymorphic residues of DRB1 alleles with a proposed function.

DRB1 allele	Amino Acid position												
						HB						HH	
								TCR			TCR		CD4
	P9	P6	P4	P4	P9	P9	P7	P7	P4/7	P4			
	9	11	13	26	37	57	67	70	71	74	77	112	140
**Susceptibility alleles**													
03:01	E	S	S	Y	N	D	L	**Q**	K	R	N	H	T
04:01	E	V	H	F	Y	D	L	**Q**	K	A	T	H	T
04:05	E	V	H	F	Y	S	L	**Q**	R	A	T	H	T
**Protective alleles**													
10:01	E	V	F	V	Y	D	L	**R**	R	A	T	H	T
11:04	E	S	S	F	Y	D	F	**D**	R	A	T	H	T
14:01	E	S	S	F	F	A	L	**R**	R	E	T	Y	T
16:02	W	P	R	F	S	D	L	**D**	R	A	T	H	A

Positions identified in bold show amino acids that are exclusive to the type 1 diabetes susceptibility or protective alleles. The function associated with each amino acid is depicted on top. P; pocket (peptide binding), HH; homodimer of heterodimer, HB; hydrogen bond to peptide, TCR; site of contact of HLA molecule to T-cell receptor. CD4; site of contact of HLA molecule to CD4.

**Table 6:** Type 1 diabetes associated polymorphic residues of DQB1 alleles with a proposed function.

DQB1 allele	Amino Acid position																		
								HH				TCR							
				HB						SB									
	P4	P4	P4/7	P6	P9	P7				P9		P7	P4	P4/7	P4	P1	P1	P1	P1
	13	26	28	30	37	47	52	53	55	57	66	67	70	71	74	85	86	89	90
**Susceptibility alleles**																			
02:01	G	**L**	S	S	I	F	L	L	L	**A**	D	I	R	K	A	L	E	T	T
03:02	G	**L**	T	Y	Y	Y	P	L	P	**A**	E	V	R	T	E	L	E	T	T
**Protective alleles**																			
03:01	A	**Y**	T	Y	Y	Y	P	L	L	**D**	E	V	R	T	E	L	E	T	T
05:03	G	**G**	T	H	Y	Y	P	Q	P	**D**	E	V	G	A	S	V	A	G	I

Positions identified in bold show amino acids that are exclusive to the type 1 diabetes positively or negatively associated alleles. The function associated with each amino acid is depicted on top. P; pocket (peptide binding), HH; homodimer of heterodimer, HB; hydrogen bond to peptide, SB; salt bridge, TCR; site of contact of HLA molecule to T-cell receptor.

**Table 7:** Type 1 diabetes associated polymorphic residues of DQA1 alleles with a proposed function.

DQA1 allele	Amino Acid position									
									CD4	
				TCR			HB			HH
	P6	P1				P6	P6/9	P9		
	11	52	55	61	64	66	69	76	129	175
**Susceptibility alleles**										
03:01	**Y**	**R**	**R**	**F**	**T**	**I**	**L**	**V**	H	**E**
05:01	**Y**	**R**	**R**	**F**	**T**	**I**	**L**	**L**	H	**K**
**Protective alleles**										
01:01	**C**	**S**	**G**	**G**	**R**	**M**	**A**	**M**	Q	**Q**
01:02	**C**	**S**	**G**	**G**	**R**	**M**	**A**	**M**	Q	**Q**
01:03	**C**	**S**	**G**	**G**	**R**	**M**	**A**	**M**	H	**Q**

Positions identified in bold show amino acids that are exclusive to the type 1 diabetes susceptibility or protective alleles. The function associated with each amino acid is depicted on top. P; pocket (peptide binding), HH; homodimer of heterodimer, HB; hydrogen bond to peptide, TCR; T-cell receptor contact site to HLA molecule, CD4; site of contact of HLA molecule to CD4.

Overall, all risk associated HLA alleles contain more susceptibility residues and protective alleles contain more protective residues. Some residues, however, might not be as critical as others. For example, HLA-DRB1 susceptibility residue E^9^ and L^67^ and resistance residue T^77^ are found in both risk and protective HLA-DRB1 alleles. In contrast, all risk HLA-DRB1 alleles contain the susceptibility epitope Q^70^ which is absent from protective alleles. Epitopes also alter the susceptibility of closely related alleles. For example, HLA-DQB1*03:01 and *03:02 have very similar amino acid sequences but differ at critical position 57, risk associated HLA-DQB1*03:02 contains alanine (A), whereas protective HLA-DRB1*03:01 contains aspartic acid (D) in the same position. In addition, risk associated HLA-DQB1*03:02 also contains the susceptibility epitope L^26^. Finally, a sequence of 10 amino acid residues could differentiate risk and protective HLA-DQA1 alleles. Epitope Y^11^R^52^R^55^F^61^T^64^I^66^L^69^V/L^76^H^129^E/K^175^ was exclusive to risk associated alleles and was not observed in any protective alleles.

### Extended risk haplotype of HLA class II susceptibility epitopes accounts for 92% of type 1 patients

We counted the number of individuals carrying an extended haplotype containing all the HLA class II risk associated epitopes in our patient-control cohort (Table 8). The epitopes we included were the ones that differentiated risk and protective alleles as described above. The vast majority of patients (167, 92%) carried at least one copy of the DRβQ^70^-DQβL^26^A^57^DQαY^11^R^52^R^55^F^61^T^64^I^66^L^69^V/L^76^ H^129^E/K^175^ epitope haplotype compared with only 27% of the control subjects. A very small percentage of patients had only the DRβ Q^70^ residue (2.9%) or only the DQβ A^57^ residue (4.1%) or none of the two (2.9%). Among control subjects the highest percentage (73%) had none of these susceptibility epitopes compared to only 8% of patients (p≤0.0001, OR 0.03).

**Table 8:** Extended haplotype of class II risk epitopes in type 1 diabetes patients and control subjects.

HLA class II Genotype	Patients (%) (N = 182)	Control subjects (%) (N = 192)	P value	OR	95% CI
DRB Q ^70^- DQB L^26^A^57^ DQA Y^11^R^52^R^55^F^61^T^64^I^66^L^69^V/L^76^H^129^E/K^175^/ X	103 (57%)	48 (25%)	<0.0001	3.8	2.5–5.9
DRB Q ^70^—DQB L^26^A^57^ DQA Y^11^R^52^R^55^F^61^T^64^I^66^L^69^V/L^76^H^129^E/K^175^/ DRB Q ^70^—DQB L^26^A^57^ DQA Y^11^R^52^R^55^F^61^T^64^I^66^L^69^V/L^76^H^129^E/K^175^	64 (35%)	3 (2%)	<0.0001	34.2	10.5–111.2
X / X (non-risk)	15 (8%)	141 (73%)	<0.0001	0.03	0.02–0.06

## Discussion

The association of class II alleles with T1D susceptibility is well documented even though the exact mechanism that confers the disease risk is yet to be fully understood. The allele frequencies of Cypriot T1D patients had not been previously reported. The vast majority of patients (79%) carried the established risk haplotypes DR3—DQ2.5 and DR4—DQ8 either in heterozygous or homozygous, or carried both haplotypes, while only 20% of the control population were carriers (p<0.001). A significant percentage (21%) of our diabetic cohort did not carry the risk haplotypes probably due to these not being present at high frequency in the Cypriot population, especially as compared to European Caucasians. In the latter, these haplotypes represent the first and second most common haplotypes respectively in contrast to ranking 87^th^ and 91^st^ in Cypriots. We thus believe that the Cypriot population represents an excellent study sample that can allow further dissection of T1D disease susceptibility. Using our highly diverse cohort of Cypriot patients, we were able to identify a new recombinant predisposing haplotype, DR4 –DQ2.3, carried by 13% of our patients, but only 4.7% of control subjects. This haplotype failed to reach significance in a large T1D Genetics Consortium investigating HLA-DR-DQ haplotypes in 607 Caucasian families and 38 Asian families [30]. A more recent study of the T1D Genetics Consortium analyzing more than 18,000 individuals of European descent also did not report this haplotype, with the authors discussing that the homogeneity of such populations may indeed limit the ability to interrogate rare alleles [31]. Further verification of the significance of the DR4 –DQ2.3 haplotype in disease susceptibility should be pursued in a larger cohort of Cypriot patients.

Recent studies have supported the significance of epitope analysis as an additional piece of the complex puzzle of deciphering autoimmune disease susceptibility [23,31,32], we thus attempted a similar analysis in our own cohort. The polymorphic residues of the HLA class II molecules are important not only for peptide binding but also interaction with the T cell receptor and CD4 as well as dimerization and stability of the heterodimer. Therefore, unlike previous studies, in our study we included residues outside of the binding pockets. Using the SKDM HLA Tool, an independent tool from ones used in previous studies, we were able to confirm the significance of a number of previously reported susceptibility and resistance epitopes, the vast majority found within the antigen-binding clefts of MHCII.

Susceptibility residue HLA-DQB A^57^ and protective counterpart consisting of aspartic acid (D) in the same position were found to have the strongest association in agreement with previous reports [23,24,31]. This aspartic acid forms a salt bridge with a conserved arginine (R) at position 76 of HLA-DQA [28], and has been correlated with protection. In contrast, the presence of a non-charged amino acid at position 57, likely incapable of forming a salt bridge, predisposes to T1D [33,34]. In addition, absence of HLA-DQB D^57^ in combination with HLA-DQA R^52^ has been associated with susceptibility [35]; the proximity of these residues to the interface of the dimer may affect the stability or structure of the dimer of heterodimers [28]. The most significantly associated HLA-DRB1 susceptibility residue identified in our study was Q^70^ not previously associated with T1D. We were able to show that this residue alone discriminated between resistance and susceptibility HLA-DRB1 alleles. In addition to Q^70^, we found residues HLA-DRβ V^11^, H^13^ and L^67^ that were previously reported to have the highest association with RA susceptibility, whereas D^70^ strongly correlated with resistance [32]. The same study identified a two amino acid epitope QA^70,74^ associated with RA susceptibility. In addition to resistance residue HLA-DRβ D^70^, we identified an alternative in the same position of the HLA-DRB1 allele, R^70^, which was actually more protective. For the different epitopes a gene dose effect was also observed. In support of these findings, previously published work has shown a dose effect in the response of insulin B chain reactive T cells, with stronger responses shown in the presence of at least one non-risk DQB D^57^ as compared to subjects lacking this epitope on both DQ alleles [36].

The importance of epitope analysis becomes apparent when considering closely related haplotypes or alleles with different risk determined by the presence of certain residues. For example, the closely related haplotypes DRB1*04:01-DQA1*03:01-DQB1*03:02 and DRB1*04:04-DQA1*03:01-DQB*03:02 differ only at positions 71 (lysine vs. arginine) and 86 (glycine vs. valine) of DRB1; however, the former is highly predisposing whereas the latter haplotype is neutral [30]. Similarly, we showed that closely related alleles HLA-DQB1*03:02, which is risk associated, and HLA-DQB1*3:01, which is protective, differ at critical positions 26 and 57. In addition, sharing of epitopes by disparate alleles may explain disease association but also disease susceptibility in the absence of high risk alleles. For example, two distinct HLA molecules not closely related but both risk associated, HLA-DQA1 *03:01 and *05:01, shared an extended haplotype of 10 amino acid residues (DQA Y^11^R^52^R^55^F^61^T^64^I^66^L^69^V/L^76^H^129^E/K^175^) all found to be significantly associated with disease susceptibility and all entirely different from all other DQA alleles suggesting that this constitutes a shared epitope for T1D. Finally, we observed that whilst the majority of our patient cohort carried the established DR3—DQ2.5 and DR4—DQ8 risk haplotypes, 13% carried one copy of the DR4—DQ2.3 haplotype and a small number of patients did not carry any of the known risk alleles. We were able to find that all patients however, even those that did not carry susceptibility haplotypes, carried identified susceptibility epitopes in their DRB1 locus and one or more in the DQA1 and DQB1 loci.

Our study reports for the first time a new haplotype, DR4 –DQ2.3 in T1D. In addition, our study lends further support to the significant role of certain HLA risk epitopes. HLA residues DRB Q^70^, DQB L^26^ and A^57^ and a 10 amino acid epitope of DQA were identified to be the most significant in discriminating risk alleles. Of our patient cohort 92% were carriers of the DQA Y^11^R^52^R^55^F^61^T^64^I^66^L^69^V/L^76^H^129^E/K^175^ with DRβ Q^70^ and DQβ L^26^A^57^, in contrast to 25% of our controls, suggesting that this extended HLA class II epitope haplotype is involved in the disease pathogenesis while other genetic factors may act as disease modifiers. Since these amino acids are implicated in functions other than antigen binding this may suggest contribution outside of the peptide groove and binding affinity to auto-antigens, to other allosteric sites that also hold important immune functions. Certainly, the contribution of either pocket or non-pocket residues to pathogenesis can only be proven by functional assays studying autoimmune TCR engagement with self-peptide/MHC. Most of the information currently available comes from studies of TCR interaction with foreign peptide however it is well-known that autoreactive T cell receptors engage peptide/MHC in configurations differently than those of pathogen responses [37,38]. Further structural data from self-reactive CD4 T cell receptors binding to self- peptide/MHC II complexes are needed to verify the implication of individual epitopes.

## Materials and methods

### Study population

A dataset of previously enrolled consented case (T1D patients) and control (individuals with no history of diabetes) subjects was used [19,39]. The case cohort consisted of 170 Greek Cypriot patients with a cut off age of disease onset set at 39 years (89 females, 81 males, mean age 11 years, age of onset: 0–8 years 59 patients, 9–13 years 60 patients, 14–39 51 patients). Patients over 40 years were considered as Latent Autoimmune Diabetes in Adults (LADA) and were not included in the final cohort. The inclusion criteria were: (i) the patient had to be of Greek-Cypriot origin, (ii) diagnosis of T1D was based on clinical (polyuria, polydipsia, weigh loss) and laboratory findings (fasting blood sugar level>125mg/dl, glycosuria, ketonuria, frequent metabolic acidosis, absent C-peptide, elevated Hb A1c and in all cases GAD antibodies), (iii) insulin treatment at onset and thereafter, (iv) in case of affected siblings only one sibling was included in the study. Information regarding gender, date of birth, ethnicity, age of onset and family history of T1D were collected for each patient. The control group consisted of 192 healthy individuals of the same ethnic descent (78 females, 114 males). The study was reviewed and approved by the Cyprus National Bioethics Committee. In the case of minors/children, a written informed consent was obtained from parents or legal guardians.

### Genotyping of HLA class II loci

Genomic DNA was extracted from whole blood with the use of commercially available QIAGEN^®^ genomic DNA extraction kit. High resolution HLA genotyping was performed by HistoGenetics at 4 classical major histocompatibility complex loci DRB1, DQA1, DQB1 and DPB1 using Next Generation Sequencing (NGS), as previously described [40].

### HLA allele frequency and epitope analysis

For allele frequency and epitope analysis the SKDM HLA Tool beta was used [41], which can test for HLA allele differences between two populations and perform amino acid analysis by retrieving amino acid sequences. Highest polymorphism is found amongst residues lining the binding pockets of HLA molecules however in this analysis both pocket and non-pocket amino acid epitopes were investigated. Once primary associations are identified other parameters are determined such as zygosity, interaction and linkage disequilibrium among amino acid epitopes of the same HLA molecule or between HLA isotypes.

The SKDM output includes the difference (Delta) in frequency between case and control alleles for a particular locus. A corresponding odds ratio (OR) and a corrected p-value are also supplied. P-values are corrected by the number of distinct alleles present in cases and controls. A list of statistically significant residues as a table denoting the alleles (Alls) where a residues is present, its position (Pos) in the alignment, the single letter alias of the amino acid (AA), whether it is associated (Assoc) with cases (+) or controls (–), a p-value, a p-value corrected (p^corr) by the number of AA interrogated and an associated odds-ratio (OR). For each zygosity comparison, the OR is calculated by Haldane’s modification of Woolf’s method: OR = [(a + ½)(d + ½)]/[(b + ½)(c + ½)], and the significance of its derivation from unity is estimated by Fisher’s exact test. HLA epitopes with a corrected P value ≤ 0.001 were defined as statistically significant. In addition, odds ratio (OR) ≥ 1.5 determined susceptibility while OR ≤ 0.5 determined resistance.

## Supporting information

S1 Table Allele frequency analysis for HLA-DRB1. The HLA-DRB1 typing of the patient and control populations. The table includes presence in the population and frequency, allele number and frequency, delta difference between the T1D and CTL population frequencies, a corrected P-value and the Odds Ratio (OR).
(DOCX)

S2 Table Allele frequency analysis for HLA-DQB1. The HLA-DQB1 typing of the patient and control populations. The table includes presence in the population and frequency, allele number and frequency, delta difference between the T1D and CTL population frequencies, a corrected P-value and the Odds Ratio (OR).
(DOCX)

S3 Table Allele frequency analysis for HLA-DQA1. The HLA-DQA1 typing of the patient and control populations. The table includes presence in the population and frequency, allele number and frequency, delta difference between the T1D and CTL population frequencies, a corrected P-value and the Odds Ratio (OR).
(DOCX)

S4 Table Allele frequency analysis for HLA-DPB1. The HLA-DPB1 typing of the patient and control populations. The table includes presence in the population and frequency, allele number and frequency, delta difference between the T1D and CTL population frequencies, a corrected P-value and the Odds Ratio (OR).
(DOCX)

S5 Table HLA-DRB1 Pocket epitopes. (DOCX)

S6 Table HLA-DQB1 pocket epitopes. (DOCX)

S7 Table HLA-DQA1 pocket epitopes. (DOCX)

S8 Table HLA-DRB1 non-pocket epitopes. (DOCX)

S9 Table HLA-DRB1 non-pocket zygosity. (DOCX)

S10 Table HLA-DQB1 non-pocket epitopes. (DOCX)

S11 Table HLA-DQB1 non-pocket zygosity. (DOCX)

S12 Table HLA-DQA1 non-pocket epitopes. (DOCX)

S13 Table HLA-DQA1 non-pocket zygosity. (DOCX)
