# Supplementary material for: Combined effect of glutamine at position 70 of HLA-DRB1 and alanine at position 57 of HLA-DQB1 in type 1 diabetes: An epitope analysis
Source: PLoS One. 2018 Mar 1;13(3):e0193684. doi: 10.1371/journal.pone.0193684 (PMC5832312; doi:10.1371/journal.pone.0193684)
Supplement: S1 Table — The HLA-DRB1 typing of the patient and control populations. The table includes presence in the population and frequency, allele number and frequency, delta difference between the T1D and CTL population frequencies, a corrected P-value and the Odds Ratio (OR). (DOCX) [file pone.0193684.s001.docx]

Supplemental Table 1. Allele frequency analysis for HLA-DRB1.

| HLA-DRB1 locus |  |  |  |  |  |  |  |  |  |  |  |
| --- | --- | --- | --- | --- | --- | --- | --- | --- | --- | --- | --- |
| Allele | Pop (T1D) | Freq (T1D) | Pop (CTL) | Freq (CTL) | Allele (T1D) | Freq (T1D) | Allele (CTL) | Freq (CTL) | Delta | p^corr | OR |
| 03:01 | 83 | 48.82% | 24 | 12.50% | 101 | 29.71% | 24 | 6.25% | 36.32% | 1.06x10^-12^ | 6.56 |
| 04:05 | 74 | 43.53% | 18 | 9.38% | 82 | 24.12% | 18 | 4.69% | 34.15% | 2.00x10^-12^ | 7.28 |
| 04:02 | 19 | 11.18% | 6 | 3.13% | 19 | 5.59% | 6 | 1.56% | 8.05% | 0.09413 | 3.69 |
| 04:01 | 12 | 7.06% | 1 | 0.52% | 12 | 3.53% | 1 | 0.26% | 6.54% | 0.02762 | 10.07 |
| 04:04 | 1 | 0.59% | 0 | 0.00% | 1 | 0.29% | 0 | 0.00% | 0.59% | 1 | 3.41 |
| 08:04 | 1 | 0.59% | 0 | 0.00% | 1 | 0.29% | 0 | 0.00% | 0.59% | 1 | 3.41 |
| 04:08 | 1 | 0.59% | 1 | 0.52% | 1 | 0.29% | 1 | 0.26% | 0.07% | 1 | 1.13 |
| 13:05 | 1 | 0.59% | 1 | 0.52% | 1 | 0.29% | 1 | 0.26% | 0.07% | 1 | 1.13 |
| 16:05 | 1 | 0.59% | 1 | 0.52% | 1 | 0.29% | 1 | 0.26% | 0.07% | 1 | 1.13 |
| 13:02 | 6 | 3.53% | 7 | 3.65% | 6 | 1.76% | 7 | 1.82% | -0.12% | 1 | 0.98 |
| 01:02 | 20 | 11.76% | 23 | 11.98% | 20 | 5.88% | 23 | 5.99% | -0.22% | 1 | 0.98 |
| 16:01 | 39 | 22.94% | 45 | 23.44% | 39 | 11.47% | 50 | 13.02% | -0.50% | 1 | 0.97 |
| 04:07 | 0 | 0.00% | 2 | 1.04% | 0 | 0.00% | 2 | 0.52% | -1.04% | 1 | 0.22 |
| 11:02 | 0 | 0.00% | 2 | 1.04% | 0 | 0.00% | 2 | 0.52% | -1.04% | 1 | 0.22 |
| 15:06 | 0 | 0.00% | 2 | 1.04% | 0 | 0.00% | 2 | 0.52% | -1.04% | 1 | 0.22 |
| 01:01 | 8 | 4.71% | 12 | 6.25% | 8 | 2.35% | 12 | 3.13% | -1.54% | 1 | 0.76 |
| 11:03 | 0 | 0.00% | 3 | 1.56% | 0 | 0.00% | 3 | 0.78% | -1.56% | 1 | 0.16 |
| 12:01 | 0 | 0.00% | 3 | 1.56% | 0 | 0.00% | 3 | 0.78% | -1.56% | 1 | 0.16 |
| 15:02 | 1 | 0.59% | 6 | 3.13% | 1 | 0.29% | 6 | 1.56% | -2.54% | 1 | 0.25 |
| 04:03 | 8 | 4.71% | 14 | 7.29% | 10 | 2.94% | 14 | 3.65% | -2.58% | 1 | 0.64 |
| 13:03 | 1 | 0.59% | 10 | 5.21% | 1 | 0.29% | 10 | 2.60% | -4.62% | 0.36263 | 0.15 |
| 15:01 | 6 | 3.53% | 16 | 8.33% | 6 | 1.76% | 16 | 4.17% | -4.80% | 1 | 0.42 |
| 13:01 | 0 | 0.00% | 11 | 5.73% | 0 | 0.00% | 11 | 2.86% | -5.73% | 0.03057 | 0.05 |
| 07:01 | 12 | 7.06% | 28 | 14.58% | 12 | 3.53% | 29 | 7.55% | -7.52% | 0.8505 | 0.46 |
| 11:01 | 8 | 4.71% | 27 | 14.06% | 9 | 2.65% | 27 | 7.03% | -9.35% | 0.1142 | 0.31 |
| 16:02 | 1 | 0.59% | 20 | 10.42% | 1 | 0.29% | 21 | 5.47% | -9.83% | 6.77x10^-4^ | 0.07 |
| 10:01 | 2 | 1.18% | 24 | 12.50% | 2 | 0.59% | 24 | 6.25% | -11.32% | 5.06x10^-4^ | 0.1 |
| 11:04 | 6 | 3.53% | 37 | 19.27% | 6 | 1.76% | 39 | 10.16% | -15.74% | 7.26x10^-5^ | 0.16 |
| 14:01 | 0 | 0.00% | 31 | 16.15% | 0 | 0.00% | 31 | 8.07% | -16.15% | 4.29x10^-8^ | 0.02 |

**Supplemental Table 1.** The HLA-DRB1 typing of the patient and control populations. The table includes presence in the population and frequency, allele number and frequency, delta difference between the T1D and CTL population frequencies, a corrected P-value and the Odds Ratio (OR).
