# Supplementary material for: Combined effect of glutamine at position 70 of HLA-DRB1 and alanine at position 57 of HLA-DQB1 in type 1 diabetes: An epitope analysis
Source: PLoS One. 2018 Mar 1;13(3):e0193684. doi: 10.1371/journal.pone.0193684 (PMC5832312; doi:10.1371/journal.pone.0193684)
Supplement: S2 Table — The HLA-DQB1 typing of the patient and control populations. The table includes presence in the population and frequency, allele number and frequency, delta difference between the T1D and CTL population frequencies, a corrected P-value and the Odds Ratio (OR). (DOCX) [file pone.0193684.s002.docx]

Supplemental Table 2. Allele frequency analysis for HLA-DQB1.

| HLA-DQB1 locus |  |  |  |  |  |  |  |  |  |  |  |
| --- | --- | --- | --- | --- | --- | --- | --- | --- | --- | --- | --- |
| Allele | Pop (T1D) | Freq (T1D) | Pop (CTL) | Freq (CTL) | Allele (T1D) | Freq (T1D) | Allele (CTL) | Freq (CTL) | Delta | p^corr | OR |
| 02:01 | 121 | 71.18% | 53 | 27.60% | 151 | 44.41% | 56 | 14.58% | 43.58% | 1.05x10^-15^ | 6.4 |
| 03:02 | 79 | 46.47% | 22 | 11.46% | 84 | 24.71% | 23 | 5.99% | 35.01% | 1.19x10^-12^ | 6.58 |
| 03:03 | 1 | 0.59% | 0 | 0.00% | 1 | 0.29% | 0 | 0.00% | 0.59% | 1 | 3.41 |
| 03:04 | 1 | 0.59% | 0 | 0.00% | 1 | 0.29% | 0 | 0.00% | 0.59% | 1 | 3.41 |
| 04:02 | 1 | 0.59% | 1 | 0.52% | 1 | 0.29% | 1 | 0.26% | 0.07% | 1 | 1.13 |
| 06:04 | 5 | 2.94% | 6 | 3.13% | 5 | 1.47% | 6 | 1.56% | -0.19% | 1 | 0.95 |
| 02:03 | 0 | 0.00% | 1 | 0.52% | 0 | 0.00% | 1 | 0.26% | -0.52% | 1 | 0.37 |
| 06:01 | 1 | 0.59% | 6 | 3.13% | 1 | 0.29% | 6 | 1.56% | -2.54% | 1 | 0.25 |
| 03:05 | 0 | 0.00% | 7 | 3.65% | 0 | 0.00% | 7 | 1.82% | -3.65% | 0.23877 | 0.07 |
| 06:02 | 0 | 0.00% | 7 | 3.65% | 0 | 0.00% | 7 | 1.82% | -3.65% | 1 | 0.07 |
| 06:03 | 0 | 0.00% | 13 | 6.77% | 0 | 0.00% | 13 | 3.39% | -6.77% | 0.00387 | 0.04 |
| 05:02 | 50 | 29.41% | 70 | 36.46% | 50 | 14.71% | 83 | 21.61% | -7.05% | 1 | 0.73 |
| 05:01 | 31 | 18.24% | 54 | 28.13% | 31 | 9.12% | 59 | 15.36% | -9.89% | 0.51537 | 0.57 |
| 05:03 | 0 | 0.00% | 31 | 16.15% | 0 | 0.00% | 31 | 8.07% | -16.15% | 2.14x10^-8^ | 0.02 |
| 03:01 | 15 | 8.82% | 84 | 43.75% | 15 | 4.41% | 91 | 23.70% | -34.93% | 2.85x10^-13^ | 0.13 |

**Supplemental Table 2.** The HLA-DQB1 typing of the patient and control populations. The table includes presence in the population and frequency, allele number and frequency, delta difference between the T1D and CTL population frequencies, a corrected P-value and the Odds Ratio (OR).
