# Supplementary material for: Combined effect of glutamine at position 70 of HLA-DRB1 and alanine at position 57 of HLA-DQB1 in type 1 diabetes: An epitope analysis
Source: PLoS One. 2018 Mar 1;13(3):e0193684. doi: 10.1371/journal.pone.0193684 (PMC5832312; doi:10.1371/journal.pone.0193684)
Supplement: S3 Table — The HLA-DQA1 typing of the patient and control populations. The table includes presence in the population and frequency, allele number and frequency, delta difference between the T1D and CTL population frequencies, a corrected P-value and the Odds Ratio (OR). (DOCX) [file pone.0193684.s003.docx]

Supplemental Table 3. Allele frequency analysis for HLA-DQA1.

| HLA-DQA1 locus |  |  |  |  |  |  |  |  |  |  |  |
| --- | --- | --- | --- | --- | --- | --- | --- | --- | --- | --- | --- |
| Allele | Pop (T1D) | Freq (T1D) | Pop (CTL) | Freq (CTL) | Allele (T1D) | Freq (T1D) | Allele (CTL) | Freq (CTL) | Delta | p^corr | OR |
| 03:01 | 111 | 65.29% | 41 | 21.35% | 126 | 37.06% | 45 | 11.72% | 43.94% | 1.76x10^-16^ | 6.84 |
| 05:01 | 94 | 55.29% | 95 | 49.48% | 114 | 33.53% | 107 | 27.86% | 5.81% | 1 | 1.26 |
| 04:01 | 1 | 0.59% | 0 | 0.00% | 1 | 0.29% | 0 | 0.00% | 0.59% | 1 | 3.41 |
| 01:10 | 0 | 0.00% | 1 | 0.52% | 0 | 0.00% | 1 | 0.26% | -0.52% | 1 | 0.37 |
| 02:01 | 12 | 7.06% | 26 | 13.54% | 12 | 3.53% | 27 | 7.03% | -6.48% | 0.46382 | 0.5 |
| 01:03 | 1 | 0.59% | 17 | 8.85% | 1 | 0.29% | 17 | 4.43% | -8.26% | 0.00124 | 0.09 |
| 01:02 | 54 | 31.76% | 83 | 43.23% | 55 | 16.18% | 98 | 25.52% | -11.47% | 0.23858 | 0.61 |
| 01:01 | 31 | 18.24% | 81 | 42.19% | 31 | 9.12% | 89 | 23.18% | -23.95% | 6.53x10^-6^ | 0.31 |

**Supplemental Table 3.** The HLA-DQA1 typing of the patient and control populations. The table includes presence in the population and frequency, allele number and frequency, delta difference between the T1D and CTL population frequencies, a corrected P-value and the Odds Ratio (OR).
