# Supplementary material for: Combined effect of glutamine at position 70 of HLA-DRB1 and alanine at position 57 of HLA-DQB1 in type 1 diabetes: An epitope analysis
Source: PLoS One. 2018 Mar 1;13(3):e0193684. doi: 10.1371/journal.pone.0193684 (PMC5832312; doi:10.1371/journal.pone.0193684)
Supplement: S4 Table — The HLA-DPB1 typing of the patient and control populations. The table includes presence in the population and frequency, allele number and frequency, delta difference between the T1D and CTL population frequencies, a corrected P-value and the Odds Ratio (OR). (DOCX) [file pone.0193684.s004.docx]

Supplemental Table 4. Allele frequency analysis for HLA-DPB1.

| HLA-DPB1 locus |  |  |  |  |  |  |  |  |  |  |  |
| --- | --- | --- | --- | --- | --- | --- | --- | --- | --- | --- | --- |
| Allele | Pop (T1D) | Freq (T1D) | Pop (CTL) | Freq (CTL) | Allele (T1D) | Freq (T1D) | Allele (CTL) | Freq (CTL) | Delta | p^corr | OR |
| 03:01 | 51 | 30.00% | 25 | 13.30% | 56 | 16.47% | 26 | 6.91% | 16.70% | 0.00306 | 2.76 |
| 02:01 | 90 | 52.94% | 74 | 39.36% | 105 | 30.88% | 82 | 21.81% | 13.58% | 0.21908 | 1.73 |
| 30:01 | 5 | 2.94% | 1 | 0.53% | 5 | 1.47% | 2 | 0.53% | 2.41% | 1 | 4.15 |
| 06:01 | 4 | 2.35% | 0 | 0.00% | 4 | 1.18% | 0 | 0.00% | 2.35% | 0.99807 | 10.19 |
| 01:01 | 3 | 1.76% | 0 | 0.00% | 3 | 0.88% | 0 | 0.00% | 1.76% | 1 | 7.88 |
| 17:01 | 12 | 7.06% | 10 | 5.32% | 12 | 3.53% | 11 | 2.93% | 1.74% | 1 | 1.34 |
| 19:01 | 1 | 0.59% | 0 | 0.00% | 1 | 0.29% | 0 | 0.00% | 0.59% | 1 | 3.34 |
| 51:01 | 1 | 0.59% | 0 | 0.00% | 1 | 0.29% | 0 | 0.00% | 0.59% | 1 | 3.34 |
| 11:01 | 1 | 0.59% | 1 | 0.53% | 1 | 0.29% | 1 | 0.27% | 0.06% | 1 | 1.11 |
| 15:01 | 0 | 0.00% | 1 | 0.53% | 0 | 0.00% | 1 | 0.27% | -0.53% | 1 | 0.37 |
| 71:01 | 0 | 0.00% | 1 | 0.53% | 0 | 0.00% | 1 | 0.27% | -0.53% | 1 | 0.37 |
| 14:01 | 17 | 10.00% | 20 | 10.64% | 18 | 5.29% | 21 | 5.59% | -0.64% | 1 | 0.94 |
| 46:01 | 0 | 0.00% | 2 | 1.06% | 0 | 0.00% | 2 | 0.53% | -1.06% | 1 | 0.22 |
| 04:01 | 92 | 54.12% | 104 | 55.32% | 112 | 32.94% | 126 | 33.51% | -1.20% | 1 | 0.95 |
| 05:01 | 1 | 0.59% | 4 | 2.13% | 1 | 0.29% | 4 | 1.06% | -1.54% | 1 | 0.36 |
| 09:01 | 2 | 1.18% | 6 | 3.19% | 2 | 0.59% | 6 | 1.60% | -2.01% | 1 | 0.42 |
| 23:01 | 1 | 0.59% | 5 | 2.66% | 1 | 0.29% | 5 | 1.33% | -2.07% | 1 | 0.3 |
| 13:01 | 8 | 4.71% | 13 | 6.91% | 8 | 2.35% | 14 | 3.72% | -2.20% | 1 | 0.68 |
| 10:01 | 5 | 2.94% | 16 | 8.51% | 5 | 1.47% | 16 | 4.26% | -5.57% | 0.80695 | 0.35 |
| 04:02 | 5 | 2.94% | 58 | 30.85% | 5 | 1.47% | 62 | 16.49% | -27.91% | 4.62x10^-12^ | 0.07 |

**Supplemental Table 4.** The HLA-DPB1 typing of the patient and control populations. The table includes presence in the population and frequency, allele number and frequency, delta difference between the T1D and CTL population frequencies, a corrected P-value and the Odds Ratio (OR).
