# Supplementary material for: Combined effect of glutamine at position 70 of HLA-DRB1 and alanine at position 57 of HLA-DQB1 in type 1 diabetes: An epitope analysis
Source: PLoS One. 2018 Mar 1;13(3):e0193684. doi: 10.1371/journal.pone.0193684 (PMC5832312; doi:10.1371/journal.pone.0193684)
Supplement: S5 Table — (DOCX) [file pone.0193684.s005.docx]

| **HLA LOCUS** | DRB1 | DRB1 | DRB1 | DRB1 | DRB1 | DRB1 | DRB1 | DRB1 | DRB1 | DRB1 | DRB1 |
| --- | --- | --- | --- | --- | --- | --- | --- | --- | --- | --- | --- |
| **Location** | 9 | 11 | 13 | 26 | 26 | 30 | 37 | 37 | 38 | 57 | 57 |
| **EPITOPE** | E | V | H | Y | F | R | N | F | A | S | A |
| **PATIENT (N=170)** | 164 | 110 | 110 | 106 | 142 | 3 | 88 | 12 | 3 | 74 | 0 |
| **CONTROL (N=192)** | 151 | 61 | 37 | 24 | 184 | 24 | 42 | 56 | 24 | 28 | 31 |
| **Pcorr. Value** | 2.4E-05 | 4.41E-8 | 8.8E-17 | 4.2E-12 | 0.02 | 0.003 | 5.0E-7 | 4.4E-6 | 0.003 | 1.5E-7 | 0.0003 |
| **OR** | 6.93 | 3.91 | 7.57 | 6.56 | 0.23 | 0.07 | 3.8 | 0.2 | 0.07 | 4.5 | 0.16 |
| **Associated alleles** | 03:01,04:05, 04:02, 04:01, 04:04, 08:04, 04:08, 13:05, 13:02, 04:07, 11:02, 11:03, 12:01, 04:03, 13:03, 13:01, 11:01, 10:01, 11:04, 14:01 | 04:05, 04:02, 04:01, 04:04, 04:08, 04:07, 04:03, 10:01 | 04:05, 04:02, 04:01, 04:04, 04:08, 04:07, 04:03 | 03:01 | 04:05, 04:02, 04:01, 04:04, 08:04, 04:08, 13:05, 16:05, 13:02, 16:01, 04:07, 11:02, 15:06, 11:03, 15:02, 04:03, 13:03, 15:01, 13:01, 07:01, 11:01, 16:02, 11:04, 14:01, | 10:01 | 03:01, 13:05, 13:02, 13:01 | 07:01, 14:01 | 10:01 | 04:05, 13:03 | 14:01 |

**Supplemental Table 5.** HLA-DRB1 pocket epitopes.

**Supplemental Table 5.** HLA-DRB1 pocket epitopes (continued).

| **HLA LOCUS** | DRB1 | DRB1 | DRB1 | DRB1 | DRB1 | DRB1 | DRB1 | DRB1 | DRB1 | DRB1 | DRB1 |
| --- | --- | --- | --- | --- | --- | --- | --- | --- | --- | --- | --- |
| **Location** | 60 | 67 | 67 | 67 | 70 | 70 | 70 | 71 | 71 | 74 | 74 |
| **EPITOPE** | H | L | I | F | Q | D | R | K | R | R | E |
| **PATIENT (N=170)** | 0 | 158 | 45 | 54 | 158 | 84 | 2 | 91 | 132 | 106 | 8 |
| **CONTROL (N=192)** | 31 | 142 | 84 | 104 | 97 | 157 | 52 | 33 | 185 | 24 | 46 |
| **Pcorr. Value** | 0.0003 | 0.0001 | 0.08 | 0.002 | 3.6E-18 | 7.4E-9 | 8.2E-12 | 3.0E-11 | 5.12E-6 | 4.2E-12 | 1.4E-5 |
| **OR** | 0.16 | 4.5 | 0.5 | 0.4 | 12.42 | 0.22 | 0.04 | 5.48 | 0.14 | 6.6 | 0.17 |
| **Associated alleles** | 14:01 | 03:01, 04:05, 04:01, 04:04, 04:08, 01:02, 04:07, 01:01, 04:03, 16:02, 10:01, 14:01 | 04:02, 16:05, 13:02, 11:02, 15:06, 12:01, 15:02, 13:03, 15:01, 13:01, 07:01 | 08:04, 13:05, 16:01, 11:03, 11:01, 11:04 | 03:01, 04:05, 04:01, 04:04, 04:08, 01:02, 04:07, 15:06, 01:01, 15:02, 04:03, 15:01 | 04:02, 08:04, 13:05, 16:05, 13:02, 16:01, 11:02, 11:03, 12:01, 13:03, 13:01, 07:01, 11:01, 16:02, 11:04 | 10:01, 14:01 | 03:01, 04:01, 13:03 | 04:05, 04:04, 08:04, 04:08, 13:05, 16:05, 01:02, 16:01, 04:07, 01:01, 12:01, 04:03, 07:01, 11:01, 16:02, 10:01, 11:04, 14:01 | 03:01 | 04:07, 04:03, 14:01 |
