# Supplementary material for: Combined effect of glutamine at position 70 of HLA-DRB1 and alanine at position 57 of HLA-DQB1 in type 1 diabetes: An epitope analysis
Source: PLoS One. 2018 Mar 1;13(3):e0193684. doi: 10.1371/journal.pone.0193684 (PMC5832312; doi:10.1371/journal.pone.0193684)
Supplement: S6 Table — (DOCX) [file pone.0193684.s006.docx]

| **HLA LOCUS** | DQB1 | DQB1 | DQB1 | DQB1 | DQB1 | DQB1 | DQB1 | DQB1 | DQB1 | DQB1 | DQB1 | DQB1 | DQB1 | DQB1 | DQB1 | DQB1 |
| --- | --- | --- | --- | --- | --- | --- | --- | --- | --- | --- | --- | --- | --- | --- | --- | --- |
| **Location** | 13 | 26 | 26 | 26 | 28 | 28 | 30 | 30 | 37 | 37 | 47 | 47 | 57 | 57 | 67 | 67 |
| **EPITOPE** | A | L | Y | G | S | T | S | H | I | Y | F | Y | A | D | I | V |
| **PATIENT (N=170)** | 17 | 161 | 17 | 78 | 121 | 140 | 121 | 81 | 121 | 136 | 121 | 140 | 160 | 17 | 122 | 139 |
| **CONTROL (N=192)** | 88 | 92 | 88 | 140 | 54 | 189 | 54 | 144 | 54 | 189 | 54 | 189 | 78 | 121 | 60 | 188 |
| **Pcorr. Value** | 1.1E-12 | 1.4E-22 | 1.1E-12 | 1.9E-5 | 1.6E-14 | 4.0E-6 | 1.6E-14 | 8.4E-6 | 1.6E-14 | 1.9E-6 | 1.6E-14 | 4.0E-6 | 1.3E-27 | 4.5E-25 | 7.7E-13 | 1.1E-5 |
| **OR** | 0.14 | 18.47 | 0.14 | 0.32 | 6.24 | 0.09 | 6.2 | 0.31 | 6.2 | 0.08 | 6.2 | 0.09 | 22.3 | 0.07 | 5.5 | 0.11 |
| **Associated alleles** | 03:04, 06:01, 03:01 | 02:01, 03:02, 03:03, 06:04, 02:03, 06:02, 06:03 | 03:04, 06:01, 03:01 | 04:02, 03:05, 05:02, 05:01, 05:03 | 02:01, 02:03 | 03:02, 03:03, 03:04, 04:02, 06:04, 06:01, 03:05, 06:02, 06:03, 05:02, 05:01, 05:03, 03:01 | 02:01, 02:03 | 06:04, 06:03, 05:02, 05:01, 05:03 | 02:01, 02:03 | 03:02, 03:03, 03:04, 04:02, 06:04, 03:05, 06:02, 06:03, 05:02, 05:01, 05:03, 03:01 | 02:01, 02:03 | 03:02, 03:03, 03:04, 04:02, 06:04, 06:01, 03:05, 06:02, 06:03, 05:02, 05:01, 05:03, 03:01 | 02:01, 03:02, 03:04, 03:05 | 03:03, 04:02, 02:03, 06:01, 06:02, 06:03, 05:03, 03:01 | 02:01, 04:02, 02:03, 06:01 | 03:02, 03:03, 03:04, 06:04, 03:05, 06:02, 06:03, 05:02, 05:01, 05:03, 03:01 |

**Supplemental Table 6.** HLA-DQB1 pocket epitopes.

**Supplemental Table 6.** HLA-DQB1 pocket epitopes (continued).

| **HLA LOCUS** | DQB1 | DQB1 | DQB1 | DQB1 | DQB1 | DQB1 | DQB1 | DQB1 | DQB1 | DQB1 | DQB1 | DQB1 |
| --- | --- | --- | --- | --- | --- | --- | --- | --- | --- | --- | --- | --- |
| **Location** | 70 | 70 | 71 | 71 | 74 | 74 | 85 | 85 | 86 | 86 | 89 | 89 |
| **EPITOPE** | R | G | K | A | A | S | L | V | E | A | T | G |
| **PATIENT (N=170)** | 166 | 77 | 121 | 77 | 121 | 78 | 165 | 82 | 165 | 78 | 165 | 82 |
| **CONTROL (N=192)** | 145 | 146 | 54 | 133 | 54 | 134 | 141 | 154 | 141 | 151 | 141 | 154 |
| **Pcorr. Value** | 1.6E-8 | 1.8E-7 | 1.6E-14 | 0.0004 | 1.6E-14 | 0.0004 | 5.2E-9 | 1.7E-8 | 5.2E-9 | 1.3E-8 | 5.2E-9 | 1.7E-8 |
| **OR** | 12.1 | 0.26 | 6.2 | 0.37 | 6.2 | 0.37 | 11.0 | 0.23 | 11.0 | 0.23 | 11.0 | 0.23 |
| **Associated alleles** | 02:01, 03:02, 03;03, 03:04, 06:04, 02:03, 06:01, 03:05, 03:01 | 06:02, 06:03, 05:02, 05:01, 05:03 | 02:01, 02:03 | 05:02, 05:01, 05:03 | 02:01, 02:03 | 04:02, 05:02, 05:01, 05:03 | 02:01, 03:02, 03:03, 03:04, 04:02, 02:03, 03:05, 03:01 | 06:04, 06:01, 06:02, 06:03, 05:02, 05:01, 05:03 | 02:01, 03:02, 03:03, 03:04, 04:02, 02:03, 03:05, 03:01 | 06:01, 06:02, 06:03, 05:02, 05:01, 05:03 | 02:01, 03:02, 03:03, 03:04, 04:02, 02:03, 03:05, 03:01 | 06:04, 06:01, 06:02, 06:03, 05:02, 05:01, 05:03 |
