# Supplementary material for: Combined effect of glutamine at position 70 of HLA-DRB1 and alanine at position 57 of HLA-DQB1 in type 1 diabetes: An epitope analysis
Source: PLoS One. 2018 Mar 1;13(3):e0193684. doi: 10.1371/journal.pone.0193684 (PMC5832312; doi:10.1371/journal.pone.0193684)
Supplement: S7 Table — (DOCX) [file pone.0193684.s007.docx]

**Supplemental Table 7.** HLA-DQA1 pocket epitopes.

| **HLA LOCUS** | DQA1 | DQA1 | DQA1 | DQA1 | DQA1 | DQA1 | DQA1 | DQA1 | DQA1 | DQA1 | DQA1 | DQA1 |
| --- | --- | --- | --- | --- | --- | --- | --- | --- | --- | --- | --- | --- |
| **Location** | 11 | 11 | 55 | 55 | 56 | 56 | 66 | 66 | 69 | 69 | 76 | 76 |
| **EPITOPE** | Y | C | R | G | R | G | I | M | L | A | V | M |
| **PATIENT (N=170)** | 165 | 82 | 165 | 82 | 133 | 82 | 165 | 82 | 165 | 82 | 133 | 82 |
| **CONTROL (N=192)** | 141 | 153 | 141 | 153 | 45 | 153 | 141 | 153 | 141 | 153 | 45 | 153 |
| **Pcorr. Value** | 4.2E-9 | 3.0E-8 | 4.2E-9 | 3.0E-8 | 1.6E-15 | 3.0E-8 | 4.2E-9 | 3.0E-8 | 4.2E-9 | 3.0E-8 | 1.6E-15 | 3.0E-8 |
| **OR** | 11.0 | 0.21 | 11.0 | 0.24 | 6.8 | 0.24 | 11.0 | 0.24 | 11.0 | 0.24 | 6.8 | 0.24 |
| **Associated alleles** | 03:01, 05:01, 04:01, 02:01 | 01:03, 01:02, 01:01 | 03:01, 05:01, 04:01, 02:01 | 01:03, 01:02, 01:01 | 03:01 | 01:03, 01:02, 01:01 | 03:01, 05:01, 04:01, 02:01 | 01:03, 01:02, 01:01 | 03:01, 05:01, 02:01 | 01:03, 01:02, 01:01 | 03:01 | 01:03, 01:02, 01:01 |
