# Supplementary material for: Combined effect of glutamine at position 70 of HLA-DRB1 and alanine at position 57 of HLA-DQB1 in type 1 diabetes: An epitope analysis
Source: PLoS One. 2018 Mar 1;13(3):e0193684. doi: 10.1371/journal.pone.0193684 (PMC5832312; doi:10.1371/journal.pone.0193684)
Supplement: S8 Table — (DOCX) [file pone.0193684.s008.docx]

| **HLA LOCUS** | DRB1 | DRB1 | DRB1 | DRB1 | DRB1 | DRB1 | DRB1 | DRB1 | DRB1 | DRB1 | DRB1 |
| --- | --- | --- | --- | --- | --- | --- | --- | --- | --- | --- | --- |
| **Location** | 10 | 31 | 33 | 40 | 58 | 73 | 73 | 77 | 77 | 96 | 96 |
| **EPITOPE** | E | V | H | Y | E | G | A | N | T | Y | Q |
| **PATIENT (N=170)** | 3 | 3 | 110 | 3 | 14 | 94 | 151 | 106 | 152 | 110 | 49 |
| **CONTROL (N=192)** | 24 | 24 | 37 | 24 | 67 | 50 | 189 | 24 | 192 | 37 | 99 |
| **Pcorr. Value** | 0.003 | 0.003 | 8.8E-17 | 0.003 | 6.2E-8 | 2.0E-6 | 0.01 | 4.2E-12 | 8.9E-5 | 8.8E-17 | 0.001 |
| **OR** | 0.07 | 0.07 | 7.6 | 0.07 | 0.17 | 3.5 | 0.14 | 6.6 | 0.02 | 7.6 | 0.38 |
| **Associated alleles** | 10:01 | 10:01 | 04:05, 04:02, 04:01, 04:04, 04:08, 04:07, 04:03 | 10:01 | 11:02, 11:03, 11:01, 11:04 | 03:01, 07:01 | 04:05, 04:02, 04:01, 04:04, 08:04, 04:08, 13:05, 16:05, 13:02, 01:02, 16:01, 04:07, 11:02, 15:06, 01:01, 11:03, 12:01, 15:02, 04:03, 13:03, 15:01, 13:01,11:01, 16:02, 10:01, 11:04, 14:01 | 03:01 | 04:05, 04:02, 04:01, 04:04, 08:04, 04:08, 13:05, 16:05, 13:02, 01:02, 16:01, 04:07, 11:02, 15:06, 01:01, 11:03, 12:01, 15:02, 04:03, 13:03, 15:01, 13:01, 07:01, 11:01, 16:02, 10:01, 11:04, 14:01 | 04:05, 04:02, 04:01, 04:04, 04:08, 04:07, 04:03 | 16:01, 15:02, 15:01, 16:02, 10:01 |

**Supplemental Table 8.** HLA-DRB1 non-pocket epitopes.

**Supplemental Table 8.** HLA-DRB1 non-pocket epitopes (continued).

| **HLA LOCUS** | DRB1 | DRB1 | DRB1 | DRB1 | DRB1 | DRB1 | DRB1 | DRB1 | DRB1 |
| --- | --- | --- | --- | --- | --- | --- | --- | --- | --- |
| **Location** | 98 | 98 | 104 | 104 | 112 | 120 | 166 | 180 | 181 |
| **EPITOPE** | K | E | S | A | Y | N | Q | L | M |
| **PATIENT (N=170)** | 147 | 114 | 147 | 114 | 0 | 110 | 3 | 110 | 14 |
| **CONTROL (N=192)** | 18 | 65 | 187 | 65 | 31 | 61 | 24 | 37 | 51 |
| **Pcorr. Value** | 0.01 | 4.3E-8 | 0.01 | 4.3E-8 | 0.0003 | 4.4E-8 | 0.003 | 8.8E-17 | 0.0005 |
| **OR** | 0.18 | 3.95 | 0.18 | 3.9 | 0.16 | 3.9 | 0.07 | 7.6 | 0.26 |
| **Associated alleles** | 03:01, 08:04, 13:05, 13:02, 01:02, 16:01, 11:02, 01:01, 11:03, 12:01, 15:02, 13:03, 15:01, 13:01, 11:01, 16:02, 10:01, 11:04, 14:01 | 04:05, 04:02, 04:01, 04:04, 04:08, 04:07, 04:03, 07:01 | 03:01, 08:04, 13:05, 13:02, 01:02, 16:01, 11:02, 01:01, 11:03, 12:01, 15:02, 13:03, 15:01, 13:01, 11:01, 16:02, 10:01, 11:04, 14:01 | 04:05, 04:02, 04:01, 04:04, 04:08, 04:07, 04:03, 07:01 | 14:01 | 04:05, 04:02, 04:01, 04:04, 04:08, 04:07, 04:03, 10:01 | 10:01 | 04:05, 04:02, 04:01, 04:04, 04:08, 04:07, 04:03 | 07:01, 10:01 |
