# Supplementary material for: Combined effect of glutamine at position 70 of HLA-DRB1 and alanine at position 57 of HLA-DQB1 in type 1 diabetes: An epitope analysis
Source: PLoS One. 2018 Mar 1;13(3):e0193684. doi: 10.1371/journal.pone.0193684 (PMC5832312; doi:10.1371/journal.pone.0193684)
Supplement: S9 Table — (DOCX) [file pone.0193684.s009.docx]

**Supplemental Table 9.** HLA-DRB1 non-pocket zygosity.

| **Locus** | **HLA-DRB1** | **HLA-DRB1** | **HLA-DRB1** | **HLA-DRB1** | **HLA-DRB1** |
| --- | --- | --- | --- | --- | --- |
| **Location** | 73 | 73 | 77 | 96 | 181 |
| **Epitope** | G | A | T | Q | M |
| **P corr value (Homozygous)** | NA | NA | 1.9E-8 | 2.1E-4 | NA |
| **OR (Homozygous)** | NA | NA | 0.01 | 0.03 | NA |
| **P corr value (Heterozygous)** | 2.5E-8 | 4.3E-6 | NA | 5.8E-4 | 2.2E-5 |
| **OR (Heterozygous)** | 3.6 | 0.09 | NA | 0.44 | 0.26 |
