# Supplementary material for: Combined effect of glutamine at position 70 of HLA-DRB1 and alanine at position 57 of HLA-DQB1 in type 1 diabetes: An epitope analysis
Source: PLoS One. 2018 Mar 1;13(3):e0193684. doi: 10.1371/journal.pone.0193684 (PMC5832312; doi:10.1371/journal.pone.0193684)
Supplement: S10 Table — (DOCX) [file pone.0193684.s010.docx]

**Supplemental Table 10.** HLA-DQB1 non-pocket epitopes.

| **HLA LOCUS** | DQB1 | DQB1 | DQB1 | DQB1 | DQB1 | DQB1 | DQB1 | DQB1 | DQB1 | DQB1 | DQB1 | DQB1 | DQB1 | DQB1 |
| --- | --- | --- | --- | --- | --- | --- | --- | --- | --- | --- | --- | --- | --- | --- |
| **Location** | 14 | 14 | 45 | 46 | 52 | 52 | 53 | 53 | 55 | 55 | 66 | 66 | 84 | 84 |
| **EPITOPE** | M | L | E | E | L | P | L | Q | L | R | D | E | Q | E |
| **PATIENT (N=170)** | 166 | 77 | 16 | 121 | 121 | 140 | 165 | 82 | 121 | 83 | 122 | 139 | 165 | 82 |
| **CONTROL (N=192)** | 152 | 133 | 84 | 54 | 54 | 189 | 141 | 154 | 54 | 155 | 60 | 188 | 141 | 154 |
| **Pcorr. Value** | 2.1E-8 | 0.0004 | 6.2E-12 | 1.6E-14 | 1.6E-14 | 4.0E-6 | 5.2E-9 | 1.7E-8 | 1.6E-14 | 1.5E-8 | 7.7E-13 | 1.1E-5 | 5.2E-9 | 1.7E-8 |
| **OR** | 9.8 | 0.37 | 0.14 | 6.2 | 6.2 | 0.09 | 10.9 | 0.23 | 6.2 | 0.23 | 5.5 | 0.11 | 10.9 | 0.23 |
| **Associated alleles** | 02:01, 03:02, 03:03, 03:04, 04:02, 06:04, 02:03, 06:01, 03:05, 06:02, 06:03, 03:01 | 05:02, 05:01, 05:03 | 03:04, 03:01 | 02:01, 02:03 | 02:01, 02:03 | 03:02, 03:03, 03:04, 04:02, 06:04, 06:01, 03:05, 06:02, 06:03, 05:02, 05:01, 05:03, 03:01 | 02:01, 03:02, 03:03, 03:04, 04:02, 02:03, 03:05, 03:01 | 06:04, 06:01, 06:02, 06:03, 05:02, 05:01, 05:03 | 02:01, 02:03 | 04:02, 06:04, 06:01, 06:02, 06:03, 05:02, 05:01, 05:03 | 02:01, 04:02, 02:03, 06:01 | 03:02, 03:03, 03:04, 06:04, 03:05, 06:02, 06:03, 05:02, 05:01, 05:03, 03:01 | 02:01, 03:02, 03:03, 03:04, 04:02, 02:03, 03:05, 03:01 | 06:04, 06:01, 06:02, 06:03, 05:02, 05:01, 05:03 |

**Supplemental Table 10.** HLA-DQB1 non-pocket epitopes (continued).

| **HLA LOCUS** | DQB1 | DQB1 | DQB1 | DQB1 | DQB1 | DQB1 | DQB1 | DQB1 | DQB1 | DQB1 | DQB1 | DQB1 |
| --- | --- | --- | --- | --- | --- | --- | --- | --- | --- | --- | --- | --- |
| **Location** | 87 | 87 | 87 | 90 | 90 | 116 | 116 | 125 | 125 | 125 | 167 | 185 |
| **EPITOPE** | L | Y | F | T | I | V | I | A | G | S | H | I |
| **PATIENT (N=170)** | 165 | 81 | 1 | 165 | 82 | 166 | 77 | 165 | 6 | 165 | 17 | 80 |
| **CONTROL (N=192)** | 141 | 136 | 26 | 141 | 154 | 151 | 133 | 140 | 32 | 133 | 88 | 28 |
| **Pcorr. Value** | 5.2E-9 | 0.0009 | 6.3E-5 | 5.2E-9 | 1.7E-8 | 1.0E-6 | 0.0004 | 2.6E-9 | 0.004 | 0.0004 | 1.1E-12 | 1.1E-9 |
| **OR** | 10.9 | 0.38 | 0.06 | 10.9 | 0.23 | 10.1 | 0.37 | 11.2 | 0.20 | 0.37 | 0.14 | 5.1 |
| **Associated alleles** | 02:01, 03:02, 03:03, 03:04, 04:02, 02:03, 03:05, 03:01 | 06:04, 05:02, 05:01, 05:03 | 06:01, 06:02, 06:03 | 02:01, 03:02, 03:03, 03:04, 04:02, 02:03, 03:05, 03:01 | 06:04, 06:01, 06:02, 06:03, 05:02, 05:01, 05:03 | 02:01, 03:02, 03:03, 03:04, 04:02, 06:04, 06:01, 03:05, 06:02, 06:03, 03:01 | 05:02, 05:01, 05:03 | 02:01, 03:02, 03:03, 03:04, 04:02, 03:05, 03:01 | 06:04, 06:01, 06:02, 06:03 | 05:02, 05:01, 05:03 | 03:04, 06:01, 03:01 | 03:02, 03:03, 04:02, 03:05 |
