# Supplementary material for: Combined effect of glutamine at position 70 of HLA-DRB1 and alanine at position 57 of HLA-DQB1 in type 1 diabetes: An epitope analysis
Source: PLoS One. 2018 Mar 1;13(3):e0193684. doi: 10.1371/journal.pone.0193684 (PMC5832312; doi:10.1371/journal.pone.0193684)
Supplement: S11 Table — (DOCX) [file pone.0193684.s011.docx]

**Supplemental Table 11.** HLA-DQB1 non-pocket zygosity.

| **Locus** | **HLA-DQB1** | **HLA-DQB1** | **HLA-DQB1** | **HLA-DQB1** | **HLA-DQB1** | **HLA-DQB1** | **HLA-DQB1** | **HLA-DQB1** | **HLA-DQB1** |
| --- | --- | --- | --- | --- | --- | --- | --- | --- | --- |
| **Location** | 14 | 45 | 55 | 87 | 87 | 116 | 125 | 125 | 185 |
| **Epitope** | M | E | R | Y | F | V | A | G | I |
| **P corr value (Homozygous)** | 2.E-7 | NA | 1.0E-9 | 3.3E-5 | NA | 1.8E-7 | 1.34E-11 | NA | NA |
| **OR (Homozygous)** | 10.9 | NA | 0.07 | 0.14 | NA | 11.1 | 18.57 | NA | NA |
| **P corr value (Heterozygous)** | 2.5E-7 | 2.0E-13 | 1.8E-7 | 7.5E-4 | 2.1E-6 | 1.3E-7 | 9.4E-9 | 1.4E-4 | 1.8E-11 |
| **OR (Heterozygous)** | 9.34 | 0.14 | 0.27 | 0.43 | 0.06 | 9.7 | 9.5 | 0.2 | 5.5 |
