# Supplementary material for: Combined effect of glutamine at position 70 of HLA-DRB1 and alanine at position 57 of HLA-DQB1 in type 1 diabetes: An epitope analysis
Source: PLoS One. 2018 Mar 1;13(3):e0193684. doi: 10.1371/journal.pone.0193684 (PMC5832312; doi:10.1371/journal.pone.0193684)
Supplement: S12 Table — (DOCX) [file pone.0193684.s012.docx]

**Supplemental Table 12.** HLA-DQA1 non-pocket epitopes.

| **HLA LOCUS** | DQA1 | DQA1 | DQA1 | DQA1 | DQA1 | DQA1 | DQA1 | DQA1 | DQA1 | DQA1 | DQA1 | DQA1 | DQA1 | DQA1 |
| --- | --- | --- | --- | --- | --- | --- | --- | --- | --- | --- | --- | --- | --- | --- |
| **Location** | 18 | 18 | 25 | 26 | 45 | 45 | 47 | 47 | 48 | 48 | 50 | 50 | 52 | 52 |
| **EPITOPE** | S | F | F | S | V | A | Q | R | L | W | L | E | R | S |
| **PATIENT (N=170)** | 165 | 82 | 13 | 133 | 165 | 82 | 133 | 82 | 165 | 82 | 115 | 82 | 163 | 82 |
| **CONTROL (N=192)** | 141 | 153 | 42 | 45 | 141 | 153 | 45 | 153 | 141 | 153 | 67 | 153 | 123 | 153 |
| **Pcorr. Value** | 4.2E-9 | 3.0E-8 | 1.5E-2 | 1.6E-15 | 4.2E-9 | 3.0E-8 | 1.6E-15 | 3.0E-8 | 4.2E-9 | 3.0E-8 | 3.2R-8 | 3.0E-8 | 3.5E-13 | 3.0E-8 |
| **OR** | 10.9 | 0.24 | 0.30 | 6.8 | 10.9 | 0.24 | 6.8 | 0.24 | 10.9 | 0.24 | 3.9 | 0.24 | 12.3 | 0.24 |
| **Associated alleles** | 03:01, 05:01, 04:01, 02:01 | 01:03, 01:02, 01:01 | 02:01, 01:03 | 03:01 | 03:01, 05:01, 04:01, 02:01 | 01:03, 01:02, 01:01 | 03:01 | 01:03, 01:02, 01:01 | 03:01, 05:01, 04:01, 02:01 | 01:03, 01:02, 01:01 | 03:01, 02:01 | 01:03, 01:02, 01:01 | 03:01, 05:01, 04:01 | 01:03, 01:02, 01:01 |

**Supplemental Table 12.** HLA-DQA1 non-pocket epitopes (continued).

| **HLA LOCUS** | DQA1 | DQA1 | DQA1 | DQA1 | DQA1 | DQA1 | DQA1 | DQA1 | DQA1 | DQA1 | DQA1 | DQA1 | DQA1 |
| --- | --- | --- | --- | --- | --- | --- | --- | --- | --- | --- | --- | --- | --- |
| **Location** | 53 | 53 | 61 | 61 | 64 | 64 | 80 | 80 | 129 | 129 | 175 | 175 | 187 |
| **EPITOPE** | R | K | F | G | T | R | S | Y | H | Q | E | Q | T |
| **PATIENT (N=170)** | 115 | 82 | 165 | 82 | 165 | 82 | 165 | 82 | 165 | 81 | 115 | 82 | 133 |
| **CONTROL (N=192)** | 67 | 153 | 141 | 153 | 141 | 153 | 141 | 153 | 147 | 142 | 67 | 153 | 45 |
| **Pcorr. Value** | 3.2E-8 | 3.0E-8 | 4.2E-9 | 3.0E-8 | 4.2E-9 | 3.0E-8 | 4.2E-9 | 300E-8 | 2.6E-7 | 2.3E-5 | 3.2E-8 | 3.0E-8 | 1.6E-15 |
| **OR** | 3.9 | 0.24 | 10.9 | 0.24 | 10.9 | 0.24 | 10.9 | 0.24 | 9.2 | 0.32 | 3.9 | 0.24 | 6.8 |
| **Associated alleles** | 03:01, 02:01 | 01:03, 01:02, 01:01 | 03:01, 05:01, 04:01, 02:01 | 01:03, 01:02, 01:01 | 03:01, 05:01, 04:01, 02:01 | 01:03, 01:02, 01:01 | 03:01, 05:01, 04:01, 02:01 | 01:03, 01:02, 01:01 | 03:01, 05:01, 04:01, 02:01, 01:03 | 01:02, 01:01 | 03:01, 04:01, 02:01 | 01:03, 01:02, 01:01 | 03:01 |
