# Supplementary material for: Combined effect of glutamine at position 70 of HLA-DRB1 and alanine at position 57 of HLA-DQB1 in type 1 diabetes: An epitope analysis
Source: PLoS One. 2018 Mar 1;13(3):e0193684. doi: 10.1371/journal.pone.0193684 (PMC5832312; doi:10.1371/journal.pone.0193684)
Supplement: S13 Table — (DOCX) [file pone.0193684.s013.docx]

**Supplemental Table 13.** HLA-DQA1 non-pocket zygosity.

| **Locus** | **HLA-DQA1** | **HLA-DQA1** | **HLA-DQA1** | **HLA-DQA1** | **HLA-DQA1** |
| --- | --- | --- | --- | --- | --- |
| **Location** | 25 | 52 | 129 | 129 | 175 |
| **Epitope** | F | R | H | Q | E |
| **P corr value (Homozygous)** | NA | 1.7E-15 | 2.7E-9 | 1.7E-5 | 1.2E-4 |
| **OR (Homozygous)** | NA | 29.9 | 13.9 | 0.11 | 42.96 |
| **P corr value (Heterozygous)** | 6.2E-4 | 1.1E-11 | 2.6E-7 | 2.6E-5 | 2.0E-8 |
| **OR (Heterozygous)** | 0.31 | 10.1 | 7.99 | 0.36 | 3.57 |
